# Should we offer preventive treatment to all carriers of *PLN* p.(Arg14del) variant? *Con:* Do no harm to asymptomatic carriers

**DOI:** 10.1007/s12471-023-01794-z

**Published:** 2023-07-25

**Authors:** Pieter A. Doevendans

**Affiliations:** 1 University Medical Centre Utrecht, Central Military Hospital, Utrecht, The Netherlands; 2 Netherlands Heart Institute, Utrecht, The Netherlands

Assuming the term ‘preventive treatment’ indicates treatment of carriers of a severe inherited disease before they develop any symptoms, early intervention may become feasible but may not be desirable. The justification for preventive treatment would have to be found in disease penetrance. If a mutation will definitely cause disease, the decision is relatively simple if the side effects of the treatment are mild or absent.

We also would have to define ‘asymptomatic’ very clearly. Let us use the phospholamban (*PLN*) p.(Arg14del) cardiomyopathy as an example, in which penetrance of the disease is highly variable, with an arrhythmogenic phenotype presenting at a younger age than the dilated cardiomyopathy phenotype [1]. Yet, many (> 50%) carriers reach the age of 75 years without ever developing symptoms severe enough to seek medical attention. In addition, we are identifying more and more carriers through screening existing datasets (Lifelines Groningen, Blood Bank Amsterdam) in whom no overt disease is found. Therefore, just treating all carriers is against the primary principle of physicians: do no harm.

In the meantime, our diagnostic skills are improving. Using electrocardiography, echocardiography and magnetic resonance imaging, we can see the initiation of a cardiomyopathy. Beautiful studies have been completed by Van der Leur et al., who employed artificial intelligence to detect early changes in the *PLN* p.(Arg14del) electrocardiogram (ECG) [2]. In addition Taha et al. showed where and how echocardiographic strain patterns are changing and should be considered as early disease parameters [3]. Furthermore, by implanting loop recorders and modernising Holter recordings, more arrhythmias can be detected.

But do these early hints always result in symptoms? Not in a 100% of carriers. However, there is a group with early disease signs but no complaints we would like to treat. Does that entail pre-emptive treatment? Probably not, as the first signs of disease are already present as indicated by changed ECG patterns, abnormal strain analysis results and nonsustained ventricular tachycardias. The path to symptoms is then relatively short.

Therefore, I propose not to treat *all* carriers with devices or drugs. However, we can start treatment in patients who show onset of cardiomyopathy, not to prevent the disease but to delay the onset of symptoms. The next problem then is to choose an appropriate evidence-based therapy. The work by Eijgenraam et al. clearly showed that in mouse models, pharmacological treatment is not effective in reducing mortality [4]. In addition, the well-designed clinical trial iPHORECAST indicated eplerenone had no effect on delaying the disease either [5]. The team from the University Medical Centre Groningen in the Netherlands clearly showed that pre-emptive pharmacological intervention is currently not a realistic option. The only alternative left is to switch to gene therapy, which has been shown to be effective in mice [6] but is not yet an option for patients, although that is likely to change within 1–2 years. At the moment, 4 US-based gene therapy companies are looking into the *PLN* p.(Arg14del) models to find a treatment or even a cure. The options are overexpressing the normal gene, reducing the endogenous expression level of *PLN* (both alleles) or cutting and pasting the chromosomal DNA. The cut-and-paste option is based on viral delivery of the CRISPR/Cas-based tools and is currently being studied. The gene editing approach disrupting the defective allele in heterozygous mice was successful, as reported by Dave et al. [7] Tenaya (San Francisco, CA, USA) recently showed that by reducing *PLN* expression in the homozygous R14Del mouse model, life expectancy increased. Alternatively, the defective gene can be corrected by introducing the missing triplet in cardiomyocytes [8–10].

Nevertheless, these therapeutic steps are experimental and not ready to be offered to patients. Hence, they are no options for asymptomatic carriers. Maybe, in contrast to the author of the *Pro* article, I consider myself a realist by nature.
